# Physician-initiated clinical study of limb ulcers treated with a functional peptide, SR-0379: from discovery to a randomized, double-blind, placebo-controlled trial

**DOI:** 10.1038/s41514-018-0021-7

**Published:** 2018-02-13

**Authors:** Hironori Nakagami, Ken Sugimoto, Takahiro Ishikawa, Taku Fujimoto, Toshifumi Yamaoka, Misa Hayashi, Eiji Kiyohara, Hiroshi Ando, Yuta Terabe, Yoichi Takami, Koichi Yamamoto, Yasushi Takeya, Minoru Takemoto, Masaya Koshizaka, Tamotsu Ebihara, Ayumi Nakamura, Mitsunori Nishikawa, Xiang Jing Yao, Hideki Hanaoka, Ichiro Katayama, Koutaro Yokote, Hiromi Rakugi

**Affiliations:** 1 Department of Health Development and Medicine, Osaka University Graduate School of Medicine, Suita, Japan; 2 Department of Geriatric and General Medicine, Osaka University Graduate School of Medicine, Suita, Japan; 3 Department of Clinical Cell Biology and Medicine, Chiba University Graduate School of Medicine, Chiba, Japan; 4 Department of Dermatology, Osaka University Graduate School of Medicine, Suita, Japan; 5 Department of Cardiology, Kasukabe Chuo General Hospital, Saitama, Japan; 6 Plastic and Reconstructive Surgery, Tokyo Nishi Tokushukai Hospital, Nishi-tokyo, Japan; 7 Department of Diabetes, Metabolism and Endocrinology, School of Medicine, International University of Health and Welfare, Otawara, Japan; 8 Department of Dermatology, Keio University School of Medicine, Tokyo, Japan; 9 Department of Pharmacy, Osaka University Hospital, Suita, Japan; 10 Department of Medical Innovation, Osaka University Hospital, Suita, Japan; 11 Clinical Research Center, Chiba University Hospital, Chiba, Japan

## Abstract

SR-0379 is a functional peptide that has wound healing effect with anti-microbial action, making it an ideal drug to prevent infection. To evaluate the safety, efficacy, and pharmacokinetics of SR-0379 for the treatment of leg ulcers, a physician-initiated, phase I/IIa, first-in-patient clinical study was designed. A multi-center, double-blind, randomized clinical study was conducted from October 2015 to September 2016. The inclusion criteria for leg ulcers were (1) diabetes or critical limb ischemia and (2) wound size <6 cm in diameter. Twelve patients were randomized into four groups and administered 0.02%, 0.1%, or 0.5% SR-0379 or placebo treatment on skin ulcers once per day for 28 days. Efficiency was evaluated by determining the rate of wound size reduction as a primary endpoint at 4 weeks after the first treatment compared with the pre-treatment wound size. As a secondary endpoint, the DESIGN-R score index, time to wound closure, and the 50% wound size reduction ratio were also evaluated. The safety of SR-0379 was evaluated during the study period. In the evaluation of efficiency, the skin ulcer reduction rates at the last evaluation were 44.73% for the 0.02% SR-0379 group, 68.25% for the 0.1% group, and 71.61% for the 0.5% group, compared with 9.95% for the placebo group. Six adverse events were reported in four patients, of which one occurred in the placebo group, and causal relationships to study drugs were denied for all six events. Treatment with SR-0379 for chronic leg ulcers was safe, well tolerated, and effective.

## Dermatology: peptide drug development for skin ulcers

Chronic leg ulcers result in substantial impairment of patient quality of life with a socioeconomic impact both in terms of medical care and missed work days. A teams led by Hironori Nakagami at Osaka University originally identified a functional peptide, SR-0379, and evaluated the safety and efficacy of SR-0379 for the treatment of leg ulcers in a physician-initiated, first-in-patient, a multi-center, double-blind, randomized clinical study. In the evaluation of efficiency, the skin ulcer reduction rates were improved for the SR-0379 treated groups in a dose-dependent manner, compared for the placebo group with no causal adverse events. Since treatment with SR-0379 for chronic leg ulcers was safe, well tolerated, and effective in this initial clinical trial, the clinical trial on next stage will be designed toward peptide drug development.

## Introduction

Chronic leg ulcers have a significant socioeconomic impact both in terms of medical care and missed work days and result in substantial impairment of patient quality of life. One in four diabetic ulcers will result in foot amputation, which diminishes quality of life and has a 3-year survival rate of only 50%.^1,2^ A diagnosis of clinical colonization, which refers to the borderline between colonization and infection, is difficult for general physicians in the clinical setting, in particular, patients with diabetes.^3^ Therefore, wound healing drugs involving anti-bacterial action exerted by anti-microbial peptides^4–6^ are ideal to avoid infections during wound care. Moreover, the functions of anti-microbial peptides are not only limited to anti-microbial action, but also exerts anti-oxidant or wound healing functions.^7–9^

We previously identified AG30/5C, a novel peptide similar to functional anti-microbial peptides.^6,7,10,11^ AG30/5C was effective for treating ulcers in pre-clinical studies in mice,^12^ and a first-in-human trial was designed as an open-label study for severe limb ulcers.^13^ Two patients met the inclusion criteria. AG30/5C was topically administered to the wound twice per day for 11 days. In terms of safety evaluation, there were no safety concerns. In both patients, ulcer size decreased after treatment (44.62% and 10.23% decrease) and further decreased after the follow-up period (73.85% and 10.23% decrease). Topical treatment with AG30/5C for severe leg ulcers was safe, well tolerated, and potentially effective.

Based on these results, we progressed to a physician-initiated clinical trial to test a modified AG30/5C peptide, named SR-0379.^14^ In a rat infected wound model, wound healing effect of SR-0379 was higher than that of fibroblast growth factor 2 (FGF2). SR-0379 also promoted angiogenesis, granulation tissue formation, and endothelial cell and fibroblast proliferation in vitro. In this study, we conducted a multi-center, double-blinded, randomized phase I/IIa clinical study to evaluate the safety, efficacy, and pharmacokinetics of SR-0379 for the treatment of leg ulcers.

## Results

### Skin irritation test (closed patch test)

Because this was a first-in-human study of SR-0379, a closed patch test was conducted using healthy men. The lower dose of SR-0379 (0.02%) for the first-in-human study was determined based on the evaluation of efficiency by pharmacological test and safety by pre-clinical test. The maximum dose of SR-0379 (0.5%) was also determined by the upper limit of SR-0379 (0.5%) for the skin stimulant test. As shown in Supplementary Table 1, only weak reactions (grade 1) were observed with 0% SR-0379 (2 after 1 h, 1 after 24 h), 0.02% SR-0379 (1 after 1 h, 1 after 24 h), 0.25% SR-0379 (2 after 1 h, 2 after 24 h), and 0.5% SR-0379 (3 after 1 h, 1 after 24 h). The skin irritation index scores for each dose of SR-0379 were within permitted levels for clinical usage. During the trial, there were no abnormal findings related to vital signs and electrocardiogram (ECG). The only adverse effect was right omalgia, but this was unrelated to SR-0379.

### Characteristics of the clinical study patients

**Fig. 1:**
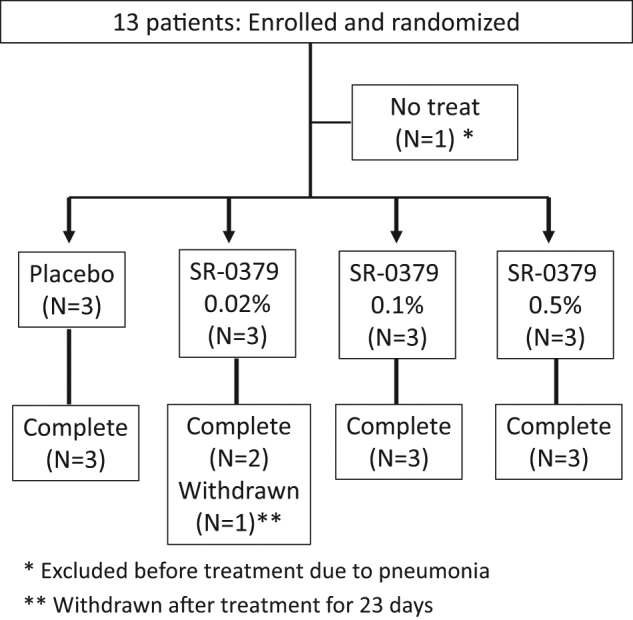
Trial profile of patients. One patient (0.02%) stopped skin ulcer treatment with SR-0379 at day 23 and was withdrawn at day 24. The patient was judged to be included in the FAS for the efficiency and safety evaluation

Thirteen patients were conducted between September 2015 and August 2016 at five hospitals in Japan. Figure 1 displays the study flow chart. All patients fulfilled the eligibility criteria. However, because one patient was excluded due to contracting pneumonia before treatment with SR-0379, 12 patients were randomized to receive treatment with the placebo or one of three doses of SR-0379 (0.02%, 0.1%, or 0.5%) for 28 days. One patient stopped skin ulcer treatment with SR-0379 (0.02%) on day 23 and was withdrawn; SR-0379 efficiency, safety, and pharmacokinetic was still evaluated at day 24. Ultimately, the patient was judged to be included in the full analysis set (FAS) for the efficiency. Thus, all 12 patients provided data for the FAS. Table 1 summarizes the characteristics of the patients. There were no marked biases in terms of sex or skin ulcer type, position, and duration among the four groups.

**Table 1:** Baseline characteristics

Characteristics	Placebo (*n* = 3)	0.02% (*n* = 3)	0.1% (*n* = 3)	0.5% (*n* = 3)
Male (%)	2 (66.7%)	2 (66.7%)	2 (66.7%)	2(66.7%)
Age (years), mean (SD)	74.3 (9.5)	63.3 (14.3)	61.0 (23.6)	76.3 (9.1)
Height (cm), mean (SD)	155.87 (18.75)	153.60 (6.59)	160.33 (13.65)	163.97 (10.1)
Weight (kg), mean (SD)	60.80 (16.20)	62.90 (12.15)	52.40 (4.10)	63.27 (1.50)
Diagnosis				
Diabetic ulcer	2 (66.7%)	2 (66.7%)	2 (66.7%)	1 (33.3%)
Ischemic ulcer	0	1 (33.3%)	1 (33.3%)	1 (33.3%)
Venous ulcer	1 (33.3%)	0	0	1 (33.3%)
Ulcer position				
Finger	2 (66.7%)	1 (33.3%)	1 (33.3%)	1 (33.3%)
Foot	1 (33.3%)	1 (33.3%)	2 (66.7%)	1 (33.3%)
Low leg	0	1 (33.3%)	0	1 (33.3%)
Duration (month)				
<3	1 (33.3%)	2 (66.7%)	1 (33.3%)	0
3 ≦ <6	0	1 (33.3%)	1 (33.3%)	1 (33.3%)
6 ≦ <12	0	0	0	1 (33.3%)
12 ≦ <36	2 (66.7%)	0	0	0
36 ≦	0	0	1 (33.3%)	1 (33.3%)

### Efficacy evaluation

**Fig. 2:**
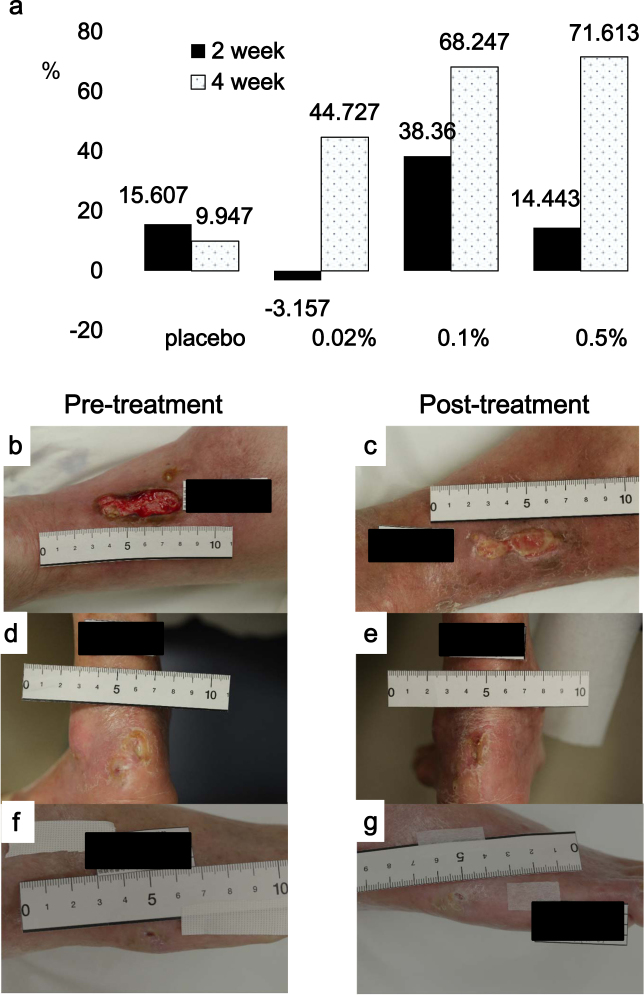
Size reduction (percent decrease) of skin ulcers with typical pictures. **a** The mean values of the percent decrease for the SR-0379-treated groups (0.02, 0.1, and 0.5%) and placebo groups are shown at 2 or 4 weeks after treatment. Pictures of the skin ulcers of four patients at pre-treatment (**b**, **d**, **f**, **h**) and post-treatment (**c**, **e**, **g**, **i**). A venous ulcer (**b**, **c**) was treated with 0.5% SR-0379 (14.84% size reduction). A heel ulcer (**d**, **e**) in a Werner Syndrome patient was treated with 0.1% SR-0379 (34.93% size reduction). An ulcer in the lateral malleolus (**f**, **g**) in a Werner Syndrome patient was treated with 0.02% SR-0379 (61.45% size reduction)

The primary endpoint was the skin ulcer rate of reduction (%) induced by SR-0379 at the final evaluation (4th week or discontinuation); these rates were 44.73 ± 41.26 (mean ± SD) for the 0.02% group (*n* = 3), 68.25 ± 28.98 for the 0.1% group (*n* = 3), and 71.61 ± 49.17 for the 0.5% group (*n* = 3) compared with 9.95 ± 65.49 for the placebo group (*n* = 3) (Fig. 2a). All reduction rate data at 2 and 4 weeks after treatment (Supplementary Fig. 1) and ulcer size at all time points (Table 2) are shown as part of the detailed analysis of each patient. At 2 weeks, only a few patients showed a marked reduction, suggesting that this time point may be too early for evaluation (Fig. 2a). In the 0.5% group, one patient demonstrated complete ulcer closure at the 2nd week, and drug administration was halted; therefore, the data reflecting 100% reduction at the 2nd week were included in the final evaluation. Additionally, another patient (0.5% group) demonstrated complete ulcer closure at 4 weeks; thus, two patients achieved a 100% reduction rate (Supplementary Fig. 1). One patient in the 0.5% group demonstrated only a 14.84% size reduction; however, ulcer depth and granulation were improved (Fig. 2b, c). One patient in the 0.1% group was diagnosed with Werner Syndrome, and his ulcer, which was on the heel, was drug-resistant; however, it demonstrated a 34.92% size reduction (Fig. 2d, e). One patient in the 0.02% group with an ulcer in the lateral malleolus was also diagnosed with Werner Syndrome. Treatment with SR-0379 resulted in a 61.45% size reduction (Fig. 2f, g).

**Table 2:** Skin ulcer size

Time point	Placebo (*n* = 3)	0.02% (*n* = 3)	0.1% (*n* = 3)	0.5% (*n* = 3)
Screening (mean ± SD)	2.720 ± 3.205	0.850 ± 0.684	0.625 ± 0.643	1.933 ± 2.260
Pre-treatment (mean ± SD)	3.540 ± 4.414	0.743 ± 0.625	2.130 ± 3.014	2.340 ± 3.358
2 weeks after treatment (mean ± SD)	2.573 ± 2.724	0.777 ± 0.783	0.320 ± 0.381	1.910 ± 3.179
Post-treatment (mean ± SD)	1.587 ± 2.724	0.563 ± 0.698	0.473 ± 0.488	1.760 ± 3.048

As a secondary endpoint (Table 3), wound closure was observed only in the 0.5% group (66.7%) after 14 days or 28 days. A significant size reduction (more than 50%) was observed in 33.3% of the placebo group and 66.7% of all SR-0379-treated groups. When local bacterial cultures were quantified, two patients (from the placebo and 0.02% SR-0379 groups) demonstrated increased bacterial quantification, and other patients exhibited no changes or decreases. As shown in Supplementary Table 2, increases were mild or moderate and were unrelated to local infection. When evaluating DESIGN-R scores, which were calculated based on six components (exudate, size, infection/inflammation, tissue granulation, necrotic tissue, and pocket size) (Supplementary Table 3), a marked decrease was observed in all SR-0379-treated group. Overall evaluation by investigators also revealed the improvement of all patients in the 0.1% and 0.5% SR-0379 groups, whereas only two patients in the placebo and 0.02% SR-0379 groups showed no improvement (worsening). Based on these results, SR-0379 treatment tends to improve ulcer status.

**Table 3:** Summary of secondary endpoints

Endpoint	Placebo (*n* = 3)	0.02% (*n* = 3)	0.1% (*n* = 3)	0.5% (*n* = 3)
Wound closure				
Yes	0 (0%)	0 (0%)	0 (0%)	2 (66.7%)
50% reduction in wound size				
Yes	1 (33.3%)	2 (66.7%)	2 (66.7%)	2 (66.7%)
Bacterial wound cultures				
Decrease	1	2	2	1
No change	1	0	1	1
Increase	1	1	0	0
Unclear	0	0	0	1^a^
DESIGN-R total score				
Pre-mean (SD)	12.7 (4.6)	9.3 (4.7)	9.0 (1.0)	12.0 (7.2)
Post-mean (SD)	9.0 (4.6)	4.3 (1.5)	5.3 (4.0)	5.3 (5.1)
Overall improvement				
Significant	1	1	1	0
Moderate	1	1	1	3
Mild	0	1	1	0
No change	0	0	0	0
Worse	1	1	0	0

^a^Bacteria were not quantified because the wound had already closed at the final visit

### Safety evaluation and pharmacokinetics

In the safety evaluation, a total of six adverse events were reported, as shown in Table 4. All adverse events were unrelated to SR-0379 treatment. Five adverse events comprised skin abrasions or ulcers in a different position unrelated to the SR-0379-treated ulcer because these patients were at high risk for skin ulcers. One serious adverse event was severe pneumonia; however, the patient had no sign of infection during treatment with 0.5% SR-0379 but became sick with pneumonia 1 month after the final SR-0379 treatment. Thus, a relationship with SR-0379 treatment was denied. There were no local adverse events such as itching, rash, or pain during treatment. Thus, there appears to be no safety concerns regarding local or systemic adverse events.

**Table 4:** List of all adverse events

Dose	Sex	Age	Events	Causality^a^	Treatment	Outcome
Mild adverse events						
0.1%	Male	41	Skin abrasion	No	No treatment	Recovery
0.1%	Male	41	Skin abrasion	No	Drug treatment	Recovery
0.1%	Male	55	New skin ulcer	No	Drug treatment	No recovery
0.5%	Female	83	New skin ulcer	No	Drug treatment	No recovery
Moderate adverse events						
Placebo	Female	84	New skin ulcer	No	Drug treatment	No recovery
Severe adverse events						
0.5%	Female	83	Pneumonia	No	Drug treatment	Death

^a^All adverse events demonstrated no drug causality

When we performed a pharmacokinetics analysis of SR-0379 in plasma, all samples from patients receiving SR-0379 treatment (0.02, 0.1, and 0.5%) had undetectable levels of SR-0379, indicating levels lower than 1 ng/mL.

## Discussion

The use of anti-microbial peptides to topically treat chronic leg ulcers demonstrates great potential based on several trials.^15,16^ We have developed novel peptide drugs to treat skin ulcers for more than 10 years, beginning with discovery during our own basic science experiments and reaching the patient bedside.^12–14,17^ The aim of this study was to evaluate the safety and efficacy of a novel functional peptide, SR-0379, for the treatment of severe limb ulcers as a first-in-patient study.

The development and approval process for wound healing drugs has yielded a high number of drug failures.^18^ In terms of study endpoints for wound healing drugs, the FDA (Food and Drug Administration) currently accepts only complete wound healing as an efficacy outcome for chronic wounds, defining complete healing as skin re-epithelialization without drainage at 2 consecutive visits 2 weeks apart by the study end.^19^ Only one drug, recombinant human PDGF-BB (becaplermin (regranex), Healthpoint Ltd, Ft. Worth, TX, USA), has obtained FDA approval for a chronic wound indication in the past 20 years.^20^ Because it is potentially difficult to show complete wound healing during a study period, an early-phase trial protocol generally includes multiple endpoints to show drug efficacy. In our case, the percent decrease in wound size after 4 weeks of treatment was defined as a primary endpoint because a previous report suggested that early changes (1 week or 4 weeks after treatment) in wound area reduction (%) were predictive of complete healing at 16 weeks.^21^ According to another report, 58% of patients demonstrating size reduction greater than the 4-week sample median achieved final healing, compared with only 9% of patients below the 4-week median, and wound area was reduced by 82% at 4 weeks in patients who achieved healing, compared with a 25% decrease at 4 weeks in patients who did not heal.^22^ In this study, the average wound size decreased in the SR-0379-treated group in a dose-dependent manner, which strongly supports the potential effects of SR-0379 on wound healing. In this study, several secondary exploratory endpoints were assessed to identify reliable biomarkers for wound healing. Among them, the DESIGN-R score was more reliable than the quantification of local bacterial cultures or the evaluation of general improvement. The Japanese Society of Pressure Ulcers originally developed this system as a tool to score the severity of pressure ulcers and monitor their healing.^23^ As it is useful to evaluate wound healing, clinical evidence for the DESIGN-R score will be required to predict wound healing together with clinical trials of SR-0379. We also sought to evaluate the management of local infection; however, the quantification of local bacterial cultures was not useful in this study because infectious wounds with clinical signs (erythema, edema, warmth, etc.) were excluded. According to guidance from the International Working Group on the Diabetic Foot (IWGDF), clinically infected diabetic foot wounds require anti-microbial therapy with systemic or topical treatment for foot care management.^5,24,25^ To evaluate the potential anti-microbial action of SR-0379, the inclusion criteria will be expanded to include wounds with signs of mild clinical infection

In the safety assessment, there were no concerns in the closed patch test, and four patients had a total of six adverse effects after SR-0379 treatment; however, a relationship with SR-0379 treatment was denied. Treatment with SR-0379 did not induce any local adverse effects (i.e., pain, rubor, or calor) or yield any systemic blood data because serum levels were very low. This suggests that SR-0379 is a very safe drug for patients. Recently, another anti-microbial peptide, LL-37, was similarly evaluated for venous leg ulcers.^26^ The study design consisted of a 4-week randomized double-blind treatment phase with LL-37 (0.5, 1.6, or 3.2 mg/mL) or placebo. Similarly, there were no safety concerns regarding local or systemic adverse events. In terms of efficiency, the healing rate constants for 0.5 and 1.6 mg/mL LL-37 were higher than those for placebo (*p* = 0.003 for 0.5 mg/mL and *p* = 0.088 for 1.6 mg/mL). However, the healing rate constants for 3.2 mg/mL LL-37 were similar to those of the placebo. An inverted dose–response efficacy was hypothesized due to increased inflammation at high doses of LL-37.^27^ In this study, bell-shaped dose–response curves for SR-0379 were not observed, suggesting the impact of SR-0379 for wound healing resembles that of similar anti-microbial peptides. Furthermore, in this study, SR-0379 treatment was administered to ulcers of patients with Werner Syndrome, which is a very rare autosomal recessive disorder^28^ caused by the WRN gene,^29^ whose protein resembles DNA helicase, that involves unusual chronic leg ulcers. One of these patients in the 0.1% SR-0379 treatment group demonstrated a 34.92% size reduction (Fig. 2d, e), and another patient in the 0.02% SR-0379 treatment group exhibited a 61.45% size reduction (Fig. 2f, g). Because ulcers associated with Werner Syndrome are reportedly drug-resistant,^30,31^ the potential mechanistic effects of SR-0379 on drug-resistant ulcers must be further analyzed.

The study limitations of this trial included the small number of patients and the short treatment period. Although there were no obvious associations between ulcer size reduction and baseline characteristics (i.e., ulcer size, duration of ulcer, and ulcer position) in this study, larger clinical studies with prolonged treatment times will be required to confirm the effects of SR-0379 until complete skin ulcer closure is achieved. It is anticipated that this academia-derived peptide drug will meet the need for a safe and effective pharmacological treatment to improve the outcomes of chronic leg ulcers.

## Methods

### Closed patch test

A closed patch test was performed with SR-0379 at Shinanozaka Clinic (Shinjuku, Tokyo, Japan); the study protocol was approved by the Shinanozaka Clinic institutional review board. The registry number is UMIN000015391, and the URL is https://upload.umin.ac.jp/cgi-open-bin/ctr_e/ctr_view.cgi?recptno=R000017479. Sixty-six men were screened after obtaining informed consent, and a total of 20 healthy men were enrolled for the patch test after obtaining informed consent between October and November 2014. The inclusion criteria for healthy men were: (1) adult age (≧20 years and ≦40 years); (2) body mass index (≧18.5 and <25.0 kg/m^2^); and (3) systolic blood pressure (SBP, ≦140 mmHg) and diastolic blood pressure (DBP, ≦90 mmHg); additionally, patients were to be within normal limits in an ECG test. The exclusion criteria were drug allergy or a history thereof, atopic dermatitis or allergic contact dermatitis, skin with dermatitis or inflammation, organ failure (heart, liver, kidney, blood), and others.

The closed patch test was performed with a Finn chamber (7 mm) using filter paper containing 0.015 mL of SR-0379 solution (0%, 0.02%, 0.1%, 0.25%, or 0.5%). The allocation was generated by a coordinator and masked for the evaluator. The test patches were applied for 48 h on the dry, non-hairy upper back after cleansing with ethanol. Results were evaluated at 1 and 24 h after removal and graded according to the International Contact Dermatitis Research Group criteria (grades 1–6). This calculation is described in Supplementary Table 1. Any adverse effects from patch testing (adhesive tape reaction, itching/flare up, angry back phenomenon, or pigment alterations) were noted.

### Preparation of a GMP-grade SR-0379 solution

GMP-grade SR-0379 peptide was generated by the American Peptide Company (Torrance, CA, USA). The peptide was dissolved in a physiological salt solution, and spray-type containers were charged with 10 mL of SR-0379 solution under GMP conditions by Nagase Medical Co. Ltd. (Itami, Hyogo, Japan). A total of 0.05 mL of SR-0379 solution was administered per spray using this container. According to the protocol, each patient received 0.25 mL of SR-0379 solution over five sprays once per day for 28 days.

### Study design and patients

The clinical trial was conducted as an investigator-initiated, phase I/IIa, randomized, double-blind, placebo-controlled trial at five hospitals (Osaka University Hospital, Osaka; Chiba University Hospital, Chiba; Asano-Kanamachi Clinic, Tokyo; Medical Plaza Shinozakieki Nishiguchi, Tokyo; and Kasukabe Chuo General Hospital, Saitama) in Japan. Twelve patients were randomized into four groups and treated with 0.02%, 0.1%, or 0.5% SR-0379 or placebo treatment. The inclusion criteria for this clinical trial were: (1) adult (age≧20 years), (2) a diagnosis of diabetic ulcer or limb ulcer (artery or venous ulcer); and (3) wound size <6 cm in diameter. Exclusion criteria is (1) infection to be cured with antibiotics, (2) deep ulcer with reaching bone tissue, (3) skin ulcer with malignant tumor, (4) severe edema around skin ulcer, (5) patients with malignant tumor, (6) patients with severe heart failure, (7) patients with severe liver, kidney, and blood dysfunction, (8) malnutrition (serum albumin ≦2 g/dL), (9) poor general condition due to severe systemic infection, (10) poor control of hyperglycemia (HbA1c ≧9.0%), (11) pregnant women, and men or women who disagreed to avoid pregnancy during clinical trial period, (12) patients who changed from an oral to an intravenous drug to regulate blood flow (i.e., prostaglandin E1, prostacyclin I2) from 2 weeks before SR-0379 or placebo treatment, (13) surgical procedure for skin ulcer from 2 weeks before SR-0379 or placebo treatment, (14) attendance to other clinical trial from 12 weeks before SR-0379 or placebo treatment, and (15) judgment as an inappropriate patient by principle investigator. The study protocol was approved by the institutional review board of each hospital. Patients gave written informed consent before enrollment.

### Randomization and masking

Twelve patients were randomized into four groups and treated with 0.02%, 0.1,% or 0.5% SR-0379 or placebo treatment. The allocation was generated by a computer program before starting the initial enrollment, and the allocation coordinator concealed the allocation information until the last patient finished. The placebo drug was indistinguishable in terms of appearance. The trial drugs were distributed to each hospital according to the allocation schedule for each patient. All study personnel and patients were masked to treatment group allocation.

### Procedures

SR-0379 (0.02%, 0.1%, and 0.5%) or placebo was topically administered after cleaning with soap once per day for 28 days. After treatment, the wound was covered with a piece of gauze. Patients who received drugs to regulate blood flow (i.e., prostaglandin E1, prostacyclin I2) did not alter their regimen starting 2 weeks before treatment through the final treatment. No topical wound healing drugs were used in combination with SR-0379 or placebo for the same skin ulcer, and the topical use of FGF2 (trafermin) was prohibited starting 2 weeks before treatment through the final treatment.

Upon study entry, clinical assessments, skin ulcer evaluations, blood and urine tests, chest radiographs, and ECGs were performed 1 week before treatment. Prior to the first treatment, clinical assessments and skin ulcer evaluations were performed, and plasma SR-0379 levels were measured at 15 and 60 min after the first treatment. After treatment, clinical assessments, skin ulcer evaluation, blood and urine tests, and ECGs were performed, and plasma SR-0379 levels were measured at 15 and 60 min after the final treatment. During the follow-up period (1 week after treatment), patients received appropriate treatment, including FGF2 (trafermin).

### Clinical study outcomes

The primary outcome was the rate of wound size reduction at 4 weeks after the first treatment compared with pre-treatment wound size. Additionally, the following secondary endpoints were measured at 4 weeks after the first treatment and compared with pre-treatment status: time to wound closure; 50% wound size reduction ratio; quantification of *Pseudomonas aeruginosa*, *Staphylococcus aureus*, MRSA, and *Streptococcus pyogenes*; and overall evaluated ulcer improvement. Ulcer size was quantified based on skin ulcer pictures according to a defined procedure using ImageJ software and calculated as the percent change. We also used the DESIGN-R score index, which is calculated based on six components (exudate, size, infection/inflammation, granulation tissue, necrotic tissue, and pocket size), as a tool to score pressure ulcer severity.^23^ Depth was excluded from the calculation. The total score was calculated by weighting the score of each item; a high score indicates greater severity. The safety of SR-0379 was evaluated during the treatment period (28 days) every 7 days and post-treatment (7 days) according to the Common Terminology Criteria for Adverse Events (version 4.0).

### Pharmacokinetics

We developed a sensitive LC-MS/MS method to study the pharmacokinetics of SR-0379 in plasma samples. SR-0379 is unstable in the plasma, and pre-treatment with EDTA and phosphoric acid (9%) inhibits its degradation. The lower limits of quantification for SR-0379 were fully validated as 1 ng/mL in plasma, with acceptable linearity, intra-assay and inter-assay precisions, and accuracy.

In this study, plasma samples obtained on day 1 (first treatment) and day 29 (final treatment) were quantified at two time points (15 and 60 min after treatment). All results were hidden to maintain the blinded study allocation until the final patient finished.

### Data availability

The data presented in this paper are tabulated in the main paper and in the Supplementary Materials are available with an appropriate material transfer agreement. All relevant data are available from the authors. The title of the registry is “Early stage clinical trial to evaluate the effect of SR-0379 on skin ulcer in safety, efficiency, and pharmacokinetics (phase I/IIa)”. The trial number is UMIN000019123, and the registry URL is https://upload.umin.ac.jp/cgi-open-bin/ctr_e/ctr_view.cgi?recptno = R000022106.

## Electronic supplementary material

supplement files

## Electronic supplementary material

**Supplementary information** accompanies the paper on the *npj Aging and Mechanisms of Disease* website (10.1038/s41514-018-0021-7).

## References

[1] Gordois A, Scuffham P, Shearer A, Oglesby A, Tobian JA (2003). The health care costs of diabetic peripheral neuropathy in the US. Diabetes Care.

[2] Reiber GE (1999). Causal pathways for incident lower-extremity ulcers in patients with diabetes from two settings. Diabetes Care.

[3] Edwards R, Harding KG (2004). Bacteria and wound healing. Curr. Opin. Infect. Dis..

[4] Jenssen H, Hamill P, Hancock RE (2006). Peptide antimicrobial agents. Clin. Microbiol. Rev..

[5] Braff MH, Gallo RL (2006). Antimicrobial peptides: an essential component of the skin defensive barrier. Curr. Top. Microbiol. Immunol..

[6] Ganz T (2003). Defensins: antimicrobial peptides of innate immunity. Nat. Rev. Immunol..

[7] Wang G, Li X, Wang Z (2016). APD3: the antimicrobial peptide database as a tool for research and education. Nucleic Acids Res..

[8] Li J (2000). PR39, a peptide regulator of angiogenesis. Nat. Med..

[9] Lai Y, Gallo RL (2009). AMPed up immunity: how antimicrobial peptides have multiple roles in immune defense. Trends Immunol..

[10] Wang G, Li X, Wang Z (2009). APD2: the updated antimicrobial peptide database and its application in peptide design. Nucleic Acids Res..

[11] Koczulla R (2003). An angiogenic role for the human peptide antibiotic LL-37/hCAP-18. J. Clin. Invest..

[12] Nakagami H (2012). Modification of a novel angiogenic peptide, AG30, for the development of novel therapeutic agents. J. Cell. Mol. Med..

[13] Nakagami, H. et al. Physician-initiated first-in-human clinical study using a novel angiogenic peptide, AG30/5C, for patients with severe limb ulcers. *Geriatr. Gerontol. Int.* **17**, 2150–2156 (2017). 10.1111/ggi.13051 28488306

[14] Tomioka H (2014). Novel anti-microbial peptide SR-0379 accelerates wound healing via the PI3 kinase/Akt/mTOR pathway. PLoS ONE.

[15] Santos R (2016). Guar gum as a new antimicrobial peptide delivery system against diabetic foot ulcers Staphylococcus aureus isolates. J. Med. Microbiol..

[16] Meier K, Nanney LB (2006). Emerging new drugs for wound repair. Expert. Opin. Emerg. Drugs.

[17] Nishikawa T (2009). Development of a novel antimicrobial peptide, AG-30, with angiogenic properties. J. Cell. Mol. Med..

[18] Maderal AD, Vivas AC, Eaglstein WH, Kirsner RS (2012). The FDA and designing clinical trials for chronic cutaneous ulcers. Semin. Cell Dev. Biol..

[19] Group, F.D.A.W.H.C.F. (2001). Guidance for industry: chronic cutaneous ulcer and burn wounds-developing products for treatment. Wound Repair Regen..

[20] Wieman TJ, Smiell JM, Su Y (1998). Efficacy and safety of a topical gel formulation of recombinant human platelet-derived growth factor-BB (becaplermin) in patients with chronic neuropathic diabetic ulcers. A phase III randomized placebo-controlled double-blind study. Diabetes Care.

[21] Lavery LA (2008). Prediction of healing for postoperative diabetic foot wounds based on early wound area progression. Diabetes Care.

[22] Sheehan P, Jones P, Caselli A, Giurini JM, Veves A (2003). Percent change in wound area of diabetic foot ulcers over a 4-week period is a robust predictor of complete healing in a 12-week prospective trial. Diabetes Care.

[23] Sanada H (2004). Reliability and validity of DESIGN, a tool that classifies pressure ulcer severity and monitors healing. J. Wound Care.

[24] Lipsky BA (2016). IWGDF guidance on the diagnosis and management of foot infections in persons with diabetes. Diabetes Metab. Res. Rev..

[25] Lipsky BA, Holroyd KJ, Zasloff M (2008). Topical versus systemic antimicrobial therapy for treating mildly infected diabetic foot ulcers: a randomized, controlled, double-blinded, multicenter trial of pexiganan cream. Clin. Infect. Dis..

[26] Gronberg A, Mahlapuu M, Stahle M, Whately-Smith C, Rollman O (2014). Treatment with LL-37 is safe and effective in enhancing healing of hard-to-heal venous leg ulcers: a randomized, placebo-controlled clinical trial. Wound Repair Regen..

[27] Niyonsaba F, Ushio H, Nagaoka I, Okumura K, Ogawa H (2005). The human beta-defensins (-1, -2, -3, -4) and cathelicidin LL-37 induce IL-18 secretion through p38 and ERK MAPK activation in primary human keratinocytes. J. Immunol..

[28] Pennisi E (1996). Premature aging gene discovered. Science.

[29] Yu CE (1996). Positional cloning of the Werner’s syndrome gene. Science.

[30] Wollina U (2004). Topical PDGF-BB results in limited healing in a patient with Werner’s syndrome and chronic leg ulcers. J. Wound Care..

[31] Yeong EK, Yang CC (2004). Chronic leg ulcers in Werner’s syndrome. Br. J. Plast. Surg..

