# Supplementary material for: Physician-initiated clinical study of limb ulcers treated with a functional peptide, SR-0379: from discovery to a randomized, double-blind, placebo-controlled trial
Source: NPJ Aging Mech Dis. 2018 Feb 13;4:2. doi: 10.1038/s41514-018-0021-7 (PMC5809414; doi:10.1038/s41514-018-0021-7)
Supplement: Supplementary file 1 — supplement files [file 41514_2018_21_MOESM1_ESM.pdf]

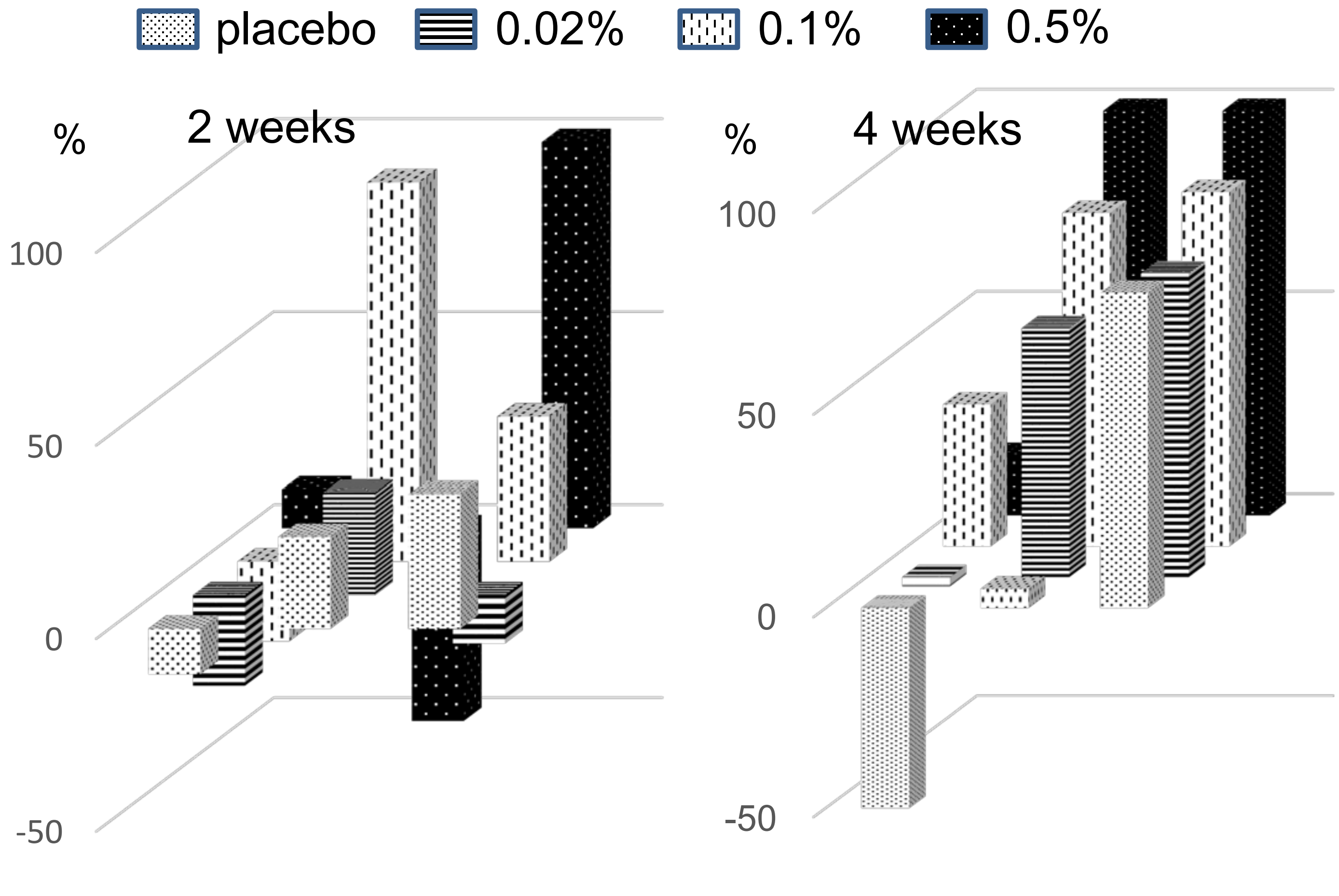

Supplement Figure 1
Size reduction (percent decrease) of skin ulcers.
The percent decrease of each patient for the SR-0379 treated (0.02%, 0.1%, and 0.5%) and placebo groups are shown at 2 or 4 weeks after treatment.

Supplemental Table 1 Evaluation of skin safety

Dose	1 hour after removal	24 hours after removal	Score[1)]	SI (%)[2)]
0%	2/20 (slight erythema)	1/20 (slight erythema)	1 (2 x 0.5)	5
0.02%	1/20 (slight erythema)	1/20 (slight erythema)	0.5 (1 x 0.5)	2.5
0.1%	0/20 (slight erythema)	0/20 (slight erythema)	0	0
0.25%	2/20 (slight erythema)	2/20 (slight erythema)	1 (2 x 0.5)	5
0.5%	3/20 (slight erythema)	1/20 (slight erythema)	1.5 (3 x 0.5)	7.5

Evaluation involved classification into 6 grades. Grade 1: no reaction, score 0; Grade 2: slight erythema, score 0.5; Grade 3: erythema, score 1; Grade 4: erythema+edema, score 2; Grade 5: erythema+edema+papule or small blister formation, score 3; Grade 6: large blister formation, score 4.

1） A strong reaction between 1 and 24 hours after removal was noted, and scoring was calculated based on the patient number for each reaction multiplied by each score.

2） SI (skin stimulative index; shown as a percent) was calculated by dividing the score by the number of patients and multiplying by 100.

Supplemental Table 2 Quantification of bacterial cultures

Score	Placebo (pre/post)	0.02% (pre/post)	0.1% (pre/post)	0.5% (pre/post)
Staphylococcus aureus				
None	1/2	1/3	0/1	0/0
Few	1/0	1/0	1/1	2/1
1+	1/0	0/0	2/1	1/1
2+	0/1	0/0	0/0	0/0
3+	0/0	1/0	0/0	0/0
Pseudomonas aeruginosa				
None	2/2	3/3	3/3	3/2
Few	0/0	0/0	0/0	0/0
1+	0/0	0/0	0/0	0/0
2+	1/1	0/0	0/0	0/0
3+	0/0	0/0	0/0	0/0
Streptococcus pyogenes				
None	3/3	3/3	3/3	3/2
Few	0/0	0/0	0/0	0/0
1+	0/0	0/0	0/0	0/0
2+	0/0	0/0	0/0	0/0
3+	0/0	0/0	0/0	0/0
**MRSA**				
None	2/3	2/2	1/1	3/2
Few	1/0	1/0	1/2	0/0
1+	0/0	0/1	1/0	0/0
2+	0/0	0/0	0/0	0/0
3+	0/0	0/0	0/0	0/0

1) Bacteria were not quantified after treatment for the 0.5% SR-0379 group because the wound of one patient had already closed at the final visit.

Supplemental Table 3 DESIGN-R score

DESIGN component weighted scores	Placebo (pre/post)	0.02% (pre/post)	0.1% (pre/post)	0.5% (pre/post)
**Depth**				
d0 None	0/0	0/0	0/1	0/0
d1 Persistent redness	0/1	0/2	0/1	0/2
d2 Extends to dermis	0/0	2/1	2/0	3/1
D3 Subcutaneous tissue	3/2	1/ 0	1/1	0/0
D4 Muscle, tendon, bone	0/0	0/0	0/0	0/0
D5 Articular, body cavity	0/0	0/0	0/0	0/0
Mean	3.0/2.3	2.3/1.3	2.3/1.3	2.0/1.3
**Exudate**				
e0 None	1/1	1/2	1/2	1/1
e1 Slight amount	1/1	2/1	1/1	1/2
e3 Moderate amount	0/1	0/0	1/0	1/0
E6 Heavy amount	1/0	0/0	0/0	0/0
Mean	2.3/1.3	0.7/0.3	1.3/0.3	1.3/0.7
**Size: area (cm^2) of a skin injury (length × width)**				
s0 None				
s3 ＜4	0/0	0/0	0/0	0/1
s6 4≦ ＜16	3/3	3/3	2/3	2/1
s8 16≦ ＜36	0/0	0/0	1/0	1/1
s9 36≦ ＜64	0/0	0/0	0/0	0/0
s12 64≦ ＜100	0/0	0/0	0/0	0/0
S15 100≦				
Mean	3.0/3.0	3.0/3.0	4.0/3.0	4.0/3.0
**Inflammation/infection**				
i0 None				
i1 Signs of inflammation	0/1	2/1	2/2	2/2
I3 Local infection	3/2	1/2	1/1	1/1
I9 Systemic impact	0/0	0/0	0/0	0/0
Mean	1.0/0.7	0.3/0.7	0.3/0.3	0.3/0.3
**Tissue granulation**				
g0 Not assessed	1/1	1/2	0/2	1/1
g1 Granulation＞90	0/0	0/1	2/0	0/1

g3 50≦Granulation＜90	0/2	0/0	0/0	1/1
G4 10≦Granulation＜50	0/0	1/0	0/0	1/0
G5 Granulation＜10	2/0	0/0	1/1	0/0
G6 No granulation	0/0	1/0	0/0	0/0
Mean	3.3/2.0	3.3/0.3	2.3/1.7	2.3/1.3
**Necrotic tissue**				
n0 None	2/3	1/3	2/3	2/3
N3 Soft necrotic tissue	1/0	2/0	1/0	0/0
N6 Hard and thick tissue	0/0	0/0	0/0	1/0
Mean	1.0/0	2.0/0	1.0/0	2.0/0
**Pocket (cm^2)**				
P0 None	2/2	3/3	3/3	2/3
P6 ＜4	1/1	0/0	0/0	1/0
P9 4≦ ＜16	0/0	0/0	0/0	0/0
P12 16≦ ＜36	0/0	0/0	0/0	0/0
P24 36≦	0/0	0/0	0/0	0/0
Mean	2.0/0	0/0	0/0	2.0/0

Six of the DESIGN components (depth was excluded) were weighted according to their relationship to healing rate, and their scores were summed to create a total DESIGN-R score ranging from 0 (healed) to 66 (greatest severity).

**DESIGN-R total score**				
Pre-mean (SD)	12.7 (4.6)	9.3 (4.7)	9.0 (1.0)	12.0 (7.2)
Post-mean (SD)	9.0 (4.6)	4.3 (1.5)	5.3 (4.0)	5.3 (5.1)

Supplemental Table 4 Patients lists with the information of ulcer and adverse events

Dose	Diagnosis	Ulcer position	Size decrease	Adverse Events
Placebo	Diabetic ulcer	finger	77.88 %	
Placebo	Diabetic ulcer	finger	4.76 %	
Placebo	Venous ulcer	foot	-52.80 %	new skin ulcer
0.02%	Diabetic ulcer	low leg	75.00 %	
0.02%	Diabetic ulcer	foot	61.45 %	
0.02%	Ischemic ulcer	finger	-2.27 %	
0.1%	Diabetic ulcer	foot	87.50 %	skin abrasion
0.1%	Ischemic ulcer	finger	82.32 %	
0.1%	Diabetic ulcer	foot	34.93 %	new skin ulcer
0.5%	Diabetic ulcer	foot	100 %	
0.5%	Ischemic ulcer	finger	100 %	
0.5%	Venous ulcer	low leg	14.84 %	pneumonia, new skin ulcer
